# Blood ACE2 Protein Level Correlates with COVID-19 Severity

**DOI:** 10.3390/ijms241813957

**Published:** 2023-09-11

**Authors:** Oksana Shevchuk, Anastasia Pak, Svitlana Palii, Yana Ivankiv, Kateryna Kozak, Mykhaylo Korda, Sandor G. Vari

**Affiliations:** 1 Department of Pharmacology and Clinical Pharmacology, I. Horbachevsky Ternopil National Medical University, 46001 Ternopil, Ukraine; pak_aniv@tdmu.edu.ua (A.P.); palij_svmy@tdmu.edu.ua (S.P.); ivankivyai@tdmu.edu.ua (Y.I.); kozakk@tdmu.edu.ua (K.K.); korda@tdmu.edu.ua (M.K.); 2 International Research and Innovation in Medicine Program, Cedars–Sinai Medical Center, Los Angeles, CA 90048, USA; vari@cshs.org

**Keywords:** COVID-19, ACE2, severity, post-COVID-19 patients, comorbidity

## Abstract

ACE2’s impact on the severity of COVID-19 is widely discussed but still controversial. To estimate its role in aspects of the main risk factors and comorbidities, we involved post-COVID-19 patients in Ternopil region (Ukraine). The recruitment period was from July 2020 to December 2021. Medical records, treatment modalities, and outcomes were recorded and analyzed. The serum human ACE2 protein was measured with Cusabio ELISA kits (Houston, TX, USA). Statistical analysis was performed with SPSS21.0 software (SPSS Inc., Chicago, IL, USA). The level of the ACE2 serum protein was significantly higher (*p* < 0.001) in patients with mild symptoms compared to a more severe course of the disease, and inversely had changed from 1 to 90 days after recovery. In patients with mild COVID-19, ACE2 levels significantly decreased over time, while among critical patients, it increased by 34.1 percent. Such results could be explained by ACE2 shedding from tissues into circulation. Loss of the membrane-bound form of the enzyme decreases the virus’ entry into cells. Our studies did not identify a sex-related ACE2 serum level correlation. The most common comorbidities were hypertension, cardiovascular diseases, respiratory diseases, and diabetes mellitus. All abovementioned comorbidities except respiratory diseases contribute to the severity of the disease and correlate with ACE2 blood serum levels.

## 1. Introduction

As of 10 May 2023, more than 765 million confirmed cases of COVID-19, including around 7 million deaths, were reported. As of 8 May 2023, a total of more than 13 billion vaccine doses have been administered (https://covid19.who.int/, accessed on 11 May 2023). Since the first cases reported in Wuhan in 2019, the virus has spread to more than 200 countries. Finally, three years later, WHO says that COVID-19 is no longer a global health emergency. However, the SARS-CoV-2 infection continues to spread. The virus is evolving and remains a global health threat but at a lower level of concern. This means that SARS-CoV-2 is still with us; however, it is no longer unusual or unexpected and does not meet the “extraordinary event” criterion. The main issue we will deal with, at least for the next couple of years, is post-COVID-19 condition, post-acute sequelae of COVID-19, or long-COVID. It is a complex heterogeneous disorder that has affected the lives of millions of people globally [1,2]. A meta-analysis (a total of more than 80,000 persons were included) demonstrated that every fourth patient experienced some clinical manifestations of long-COVID [3,4].

The clinical spectrum of SARS-CoV-2 infection ranges from asymptomatic disease to critical illness and death [5,6]. Acute respiratory distress syndrome (ARDS) and respiratory failure are the leading causes of death, but damage to other organs and systems, including the heart, liver, and kidney, also contribute to mortality rates, especially in patients with comorbidity [7,8,9,10]. The virus spreads primarily through the respiratory tract, and lymphopenia and cytokine storms have been observed in severely ill patients [11,12]. The main entry gate for SARS-CoV-2—Angiotensin Converting Enzyme 2 (ACE2)—was identified quite fast [13,14]. ACE2 receptors play an important role in the binding of viruses and are located on the surface of many cells including respiratory epithelial and endothelial cells [14,15,16]. These receptors enable entry of the virus into target cells and may be linked to more severe progression of the disease [15,17]. It became an extremely important problem to study, because among the most common comorbidities associated with a severe course of the disease are hypertension and other cardiovascular diseases (CVDs) [5,6,18]. The first-line drugs for hypertension treatment are ACE inhibitors (ACEi, -prils) and Angiotensin II Receptor Blockers (ARBs, -sartans), which are clearly established to increase ACE2 levels [19] and could contribute to COVID-19 severity. ACE and ACE2 are essential parts of the renin-angiotensin system (RAS), which maintains blood pressure and electrolyte balance and has been implicated in the pathogenesis of ARDS in COVID-19 patients. Due to that fact, at the beginning of the pandemic, controversy was observed regarding the impact of common antihypertensive treatments—ACEi and ARBs—on the severity of the SARS-CoV-2 infection. Potential benefits or harm were discussed very intensively. The virus has a high affinity to the ACE2 receptors, and its expression is thought to be upregulated in ACEi users [20,21,22]. The increased expression of ACE2 may facilitate infection with COVID-19 and increase the viremia. One more important question arises: Does hypertension itself or ACEi use impact the severity of COVID-19? The main risk factors that contribute to the severity course of coronavirus diseases are described in many studies. In addition to pre-existing comorbidities such as hypertension, diabetes mellitus, and respiratory and cardiovascular diseases, factors such as old age, male sex, occupation (e.g., healthcare workers due to higher exposed viral load and increased viral exposure time), and obesity are of the most interest [18,23,24,25,26,27]. It could be explained by the fact that due to prolonged life-long pharmacotherapy and pathophysiological changes, the presence of ACE2 is considerably increased in such patients [19]. Therefore, ACE2 is at the core of COVID-19 research, and the aim of our study was to examine its role in the severity and outcomes of coronavirus infection in regard to aspects of the main risk factors and comorbidities.

## 2. Results

Among 577 involved persons, one-third were healthcare workers—197 (34.1 percent). Patients with mild COVID-19 (Home Quarantined with Mild symptoms (HQM) group) represented almost half of the cohort—263 (45.6 percent). The Hospitalized with Moderate course (HMO) group included 215 individuals (37.3 percent) and the Hospitalized oxygen-dependent patients with Severe symptoms (HSV) group included 82 individuals (14.2 percent). Only 17 (2.9 percent) Hospitalized Critical patients in ICU departments with artificial ventilation (HCR) persons, who survived after time in the intensive care unit (ICU), were involved.

The numbers, represented in Table 1, correspond to the regular COVID-19 incidence rate and severity observed in Ukraine during the pandemic period in 2020–2021, during the Delta wave of the pandemic (Table 1).

The level of ACE2 serum protein was significantly higher (*p* < 0.001) in patients with a mild course of the disease compared to a more severe course, and even compared to seronegative clinically healthy persons (Table 2).

Severity. The serum level of ACE2 significantly decreased proportionally to COVID-19 severity. Its concentration was lower in HCR by 27.6 percent compared to HSV by 29.4 percent, and in HMO by 19.8 percent compared to HQM (*p* < 0.001). The concentration of ACE2 was higher in HMO [7.37 (5.75; 9.32) vs. 6.49 (5.28; 7.25) ng/mL], compared to the HSR group. Moreover, the serum ACE2 in the HQM group was significantly higher than in the control group by 16.3 percent (*p* < 0.001).

Sex difference. We observed a female prevalence (66.9 percent) in our cohort and there was no sex difference in ACE2 blood concentration among the observed groups. However, the level of indices was different according to the severity of COVID-19 as well as in the general cohort (Table 3).

Post-COVID-19 time period. In patients with mild COVID-19, the level of ACE2 significantly decreased over time, while among critical patients who survived, we observed the opposite situation—it increased by 34.1 percent in the abovementioned time frame (Table 4). In other groups, the tendency was the same.

**Figure 1:**
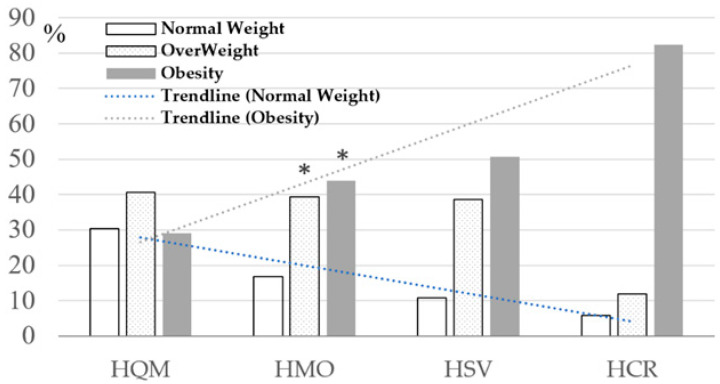
The distribution of patients according to severity of COVID-19 and BMI index (χ^2^ = 61.23; *p* < 0.001, χ^2^—Pearson Chi-square test). *—The indices were significantly different compared to HQM group, *p* < 0.05. HQM—Home Quarantined with Mild COVID-19 course, HMO—Hospitalized with Moderate course, HSV—Hospitalized oxygen-dependent patients with Severe symptoms, HCR—Hospitalized Critical patients in ICU departments with artificial ventilation.

BMI (body-mass index). We found that the proportion of patients with overweight and obesity increased directly proportionally from the HQM group to the HCR group. Among patients with mild COVID-19, 35.5 percent (93) had normal BMI, 36.3 percent (95) were overweight, and 26.3 percent (69) were obese; while in the HCR group, only 5.9 percent had a normal weight, 11.7 percent were overweight, and 82.4 percent were obese (Figure 1).

Detailed anthropometric data are presented in Table 5.

It is obvious that body weight and BMI were significantly higher in critical and oxygen-dependent patients compared to those with mild coronavirus diseases. The average body mass in the HQM group was 76.02 ± 17.31 kg, while in the HCR group, it was 93.41 ± 15.40 kg, and BMI increased from 27.17 ± 5.68 to 32.80 ± 4.91, respectively.

Among HQM patients, the ACE2 protein level was almost the same and did not differ, as well as for the severe and critical patients. However, in the HMO group, the ACE2 level was higher by 33.7 percent in obese patients compared to those with normal weight. The ACE2 serum protein level was significantly higher in all weight category patients (normal, overweight, and obese) in the HQM group compared to HMO and HSV patients (*p* < 0.05).

**Figure 2:**
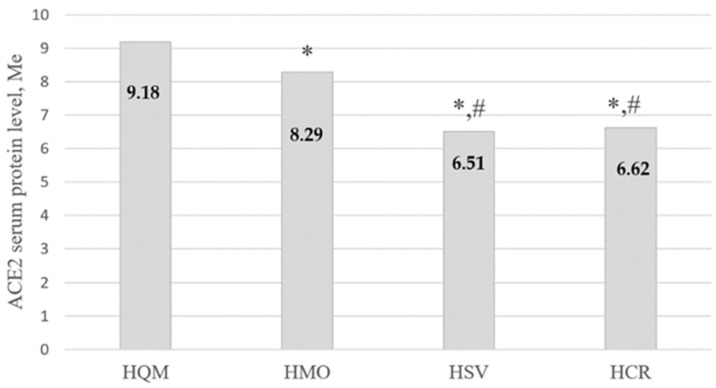
ACE2 serum protein levels in obese patients (Median, ng/mL) in different COVID-19 severity groups. *—significantly different compared to HQM group, *p* < 0.05. #—significantly different compared to HMO group, *p* < 0.05. HQM—Home Quarantined with Mild COVID-19 course. HMO—Hospitalized with Moderate course. HSV—Hospitalized oxygen-dependent patients with Severe symptoms. HCR—Hospitalized Critical patients in ICU departments with artificial ventilation.

Data for obese persons are presented in Figure 2.

Pre-existing comorbidity. In the involved cohort, the most common comorbidities were hypertension (246 patients or 42.8 percent), cardiovascular diseases (215 or 37.3 percent), respiratory diseases (73 or 12.5 percent), and diabetes mellitus (59 or 10.2 percent).

Hypertension and cardiovascular diseases. Significantly different ACE2 serum protein levels in patients with different COVID-19 severity were observed only in HQM patients with high blood pressure (absent (n = 150), 9.09 [7.83; 9.71] vs. present (n = 50), 9.40 [8.84; 10.70]) and CVDs (absent (n = 119), 9.09 [8.36; 9.84] vs. present (n = 36), 9.53 [8.36; 10.44]). We estimated a statistical difference between groups absent and present for the Mann–Whitney U test. The multivariate analysis demonstrates the correlation between cardiovascular diseases (except hypertension) and ACE2 level (8.54 [6.62; 9.45] without vs. 7.15 [5.62; 9.39] ng/mL in case of presence).

Respiratory diseases. The presence of respiratory diseases did not affect the ACE2 serum protein level.

Diabetes mellitus (DM). Most of the patients in the cohort had DM type II, and ACE2 levels were significantly higher in all examined groups compared to patients with normal glycemia in the post-COVID-19 period (Table 6). This could be explained by the known fact that Metformin, the cornerstone of treatment of DM type II, also increases ACE2 levels.

## 3. Discussion

The results of our study demonstrated that the concentration of the blood ACE2 protein is reversibly proportional to the severity of COVID-19, time-dependent (in the time frame of 0 to 90 days since the last negative PCR), and normalized with the time flow. We also identify the correlation of this index with age, history of present diseases, overweight, and obesity.

The severity of the outcome of COVID-19 is greatly influenced by various comorbidities and an unhealthy lifestyle. Since the beginning of the COVID-19 pandemic, the use of ACE inhibitors and ARBs in hypertensive patients with COVID-19 has been controversial due to the proven role of RAS in regulating blood pressure mechanisms and the role of ACE2 receptors as an entry gate to SARS-CoV-2 [28]. Suppression of the renin-angiotensin-aldosterone system is therefore a key strategy in the treatment of chronic cardiovascular and renal disease and is achieved by the administration of ACEi, ARBs, and mineralocorticoid receptor antagonists, alone or in combination.

RAS operates via the classic ACE/Angiotensin (Ang) II/Ang II type 1(AT1) receptor axis and the non-classical ACE2/Ang 1–7/Mas receptor (MasR) axis. The classical pathway is associated with the impairment of respiratory conditions, while non-classical plays a protective role in COVID-19 complications as ARDS [20,29]. ACE2 as a carboxypeptidase converts the decapeptide angiotensin I (AngI) to Ang (1–9) and the octapeptide Ang II to Ang (1–7) [30]. ACE2 possesses vasodilatory, anti-inflammatory, and anti-fibrotic effects and is the main counter-balancer for the ACE/AngII/ AT1R pathway. It has a regulatory effect on the heart, kidney, lung, and gastrointestinal tracts and regulates the homeostasis of amino acids, the expression of peptides, and local innate immune responses in the gut [31]. Bastolla et al. suggest that the main functional role of ACE2 may consist of reversing the inflammation process [28]. The roles of ACE and ACE2 have been investigated in an animal model of ARDS. ACE activity was enhanced in ARDS, whereas ACE2 activity was reduced. It correlated with enhanced levels of Ang II and reduced levels of Ang (1–7) [31]. Genetic ACE2 deficiency is one more aspect that can exacerbate the COVID-19 lung injury [32,33]. It was found that the higher the expression of ACE in the ACE I/D gene polymorphism, the more severe the onset and severity of ARDS [34]. So, ACE2 genetic polymorphism also contributes to pathogenesis. 

Our results demonstrate that post-COVID-19 patients with mild symptoms have higher ACE2 serum levels compared to those with severe infection. This is reflected in the studies of other researchers [28,35]. Our analysis strongly suggests that low serum ACE2 levels are a negative prognostic factor in the susceptibility to infection. Maza et al. also observed that high levels of serum ACE2 correlate with lower susceptibility to infection [36]. This phenomenon could be explained by the ACE2 shedding from the tissue into circulation [37]. ADAM metallopeptidase domain 17 (ADAM17), also called the tumor necrosis factor-alpha converting enzyme (TACE), and transmembrane protease serine 2 (TMPRSS2) catalyzes ACE2 shedding from the tissue into circulation. ACE2 shedding (ACE2 anchors on the cell surface are not stable and can be shed from the membrane) might reduce ACE2 surface receptor expression. It finalizes by producing soluble ACE2, resulting in the loss of the membrane-bound form of the enzyme. That is why a higher level of soluble ACE2 in the serum demonstrates a lower ability to bind the virus and enter the cell [38,39].

Membrane-bound ACE2 is the main cellular receptor of SARS-CoV-2, and it is expected that an increase in its level may enhance the infection. Nevertheless, ACE2 plays an important physiological role by downregulating the pro-inflammatory peptides, Ang II and bradykinin, and through this action, it protects the lungs from acute inflammation. Therefore, it was proposed by several authors that higher levels of ACE2 may alleviate the severity of SARS-CoV-2 infection. This hypothesis is supported by our results. However, our study is limited due to the lack of reflection on the levels and activity of the mem-brane-bound ACE2 receptor in pulmonary tissues or in other solid tissues.

Our studies did not identify any sex-related correlation of ACE2 serum level. As we know, the ACE2 encoding gene is located on the X chromosome [40], and some studies have found a higher mortality level among men [40,41,42]. This could be explained by the higher number of involved females (67 percent of the cohort). Women have increased serum ACE2 compared to men, and women seem to have milder symptoms of COVID-19. In addition, the tendency toward lower levels of soluble ACE2 is observed in the aging population [43]. ACE2 levels are low in both sexes up to the age of 12 where it increases to a greater extent in males [35]. Also, hypertension and heart failure are likely associated with higher levels of serum ACE2 activity in men compared to women [40]. Radzikowska’s et al. investigation with RNA sequencing of the expression and co-expression of ACE2 and related genes suggests altered expression of those receptors regarding age, gender, obesity, and smoking, as well as with the disease status [44]. However, Li et al. did not find any difference in ACE2 expression levels between males and females and between younger (ages ≤ 49 years) and older (ages > 49 years) persons across 31 normal human tissues [45]. So, this question requires more profound studies involving larger populations.

Alcohol consumption, opioids, and smoking were reported among factors that could affect ACE2 upregulation and expression [46,47,48,49]. The SARS-CoV-2 pandemic had a profound negative impact on society, the economics, and the healthcare functioning of most countries and it was reflected in behavior and the abovementioned habits. Based on different surveys, an increase in the consumption of alcohol, stimulant drinks, illegal substances, and pharmaceuticals prescribed to treat anxiety, depression, and sleep changes was seen [50,51]. Smoking is associated with poor COVID-19 outcomes [52,53]. Our study did not include those aspects; among the involved cohort, 20 persons reported smoking in anamnesis: 13 persons in the HQM group, 7 in HMO, and 3 in the HSV group (3.7–4.9%). The comparative analysis demonstrated no statistical difference depending on the presence or absence of smoking. This could be explained by the low number of patients and serves as a limitation of the study.

ACE2 levels and SARS-CoV-2 infection severity correlate with BMI and obesity. More fat was associated with more severe COVID-19 progress. There could be many reasons for the increased susceptibility to COVID-19 among the obese and diabetics including, for instance, impaired immune response in these patient groups. Data are based on the measurements of serum protein levels of ACE2 in 5457 individuals. Adipose tissue contains as many ACE2 receptors as pulmonary tissues, and it was found that ACE2 expression in adipose tissue of mice is increased by a high-fat diet [54]. Obesity, as well as DM, is associated with chronic low-grade inflammation that can aggravate systemic inflammation and influence the outcome of COVID-19.

We have found that the soluble ACE2 level was higher in patients with DM and should play a protective role, but it was not in this case. Clinical data by Prof. M. Hrebenyk demonstrated poor outcomes for COVID-19 patients with DM [55]. Such controversy could be explained by the late COVID-19 diagnosis in patients with DM and more severe endothelial dysfunction, caused by comorbidity. ACE2 is localized in the endothelial cells and smooth muscle cells of the cardiovascular tissue, kidney, and skin. So, this means it is a ubiquitous enzyme due to its location in vessels throughout the whole body.

Patients with isolated DM postponed admission and/or physician’s consultation and diagnosis of COVID-19 in patients the latest compared to other categories of patients (Table 7) (Hrebenyk et al. [55], article is in the press).

## 4. Materials and Methods

Post-COVID-19 patients in the period of 1–90 days after the last negative PCR test were involved in the study. Recruitment was performed in healthcare institutions of Ternopil region (Ukraine) between 15 July 2020, and 28 December 2021. Inclusion criteria included a positive PCR test for SARS-CoV-2, the availability of medical records and collected vein blood samples, and signed informed consent. Medical records, clinical manifestations, blood tests, BMI (BMI = weight (in kg)/ height^2^ (in m^2^)), treatment modalities, and outcomes were recorded and analyzed. The final number of enrolled patients was 577, including 386 (66.9 percent) females and 191 (33.1 percent) males, with an average age of 50.63 ± 13.08 years. According to the severity of COVID-19, all patients were divided into the following groups:

- HQM—**H**ome **Q**uarantined with **M**ild disease course.
- HMO—**H**ospitalized with **Mo**derate course.
- HSV—**H**ospitalized oxygen-dependent patients with **SeV**ere symptoms.
- HCR—**H**ospitalized **Cr**itical patients in ICU departments with artificial ventilation.

As a comparison group, we used 30 seronegative patients with negative PCR tests for SARS-CoV-2 and without any symptoms of respiratory illnesses at the time of examination.

Measurements of human ACE2 protein in blood serum were performed with Cusabio ELISA Kits (Houston, TX, USA). 

Statistical analysis was performed with SPSS 21.0 software (SPSS Inc., Chicago, IL, USA) using univariate and multivariate tests (Chi-square, Fisher’s exact test, Mann–Whitney U, one-way ANOVA, and Kruskal–Wallis ANOVA tests). To present quantitative variables, the arithmetical average (Mean) and (±SD) standard deviation (normal distribution) or the median (Me) and [Lq; Uq] interquartile range (different from normal distribution) were calculated (Shapiro–Wilk test for normality). *p* < 0.05 was considered statistically significant.

## 5. Conclusions

The concentration of the blood ACE2 protein is time-dependent and normalized with the time flow after the last negative PCR test. We did not find any sex differences in the indices, which could be explained by lower numbers of critical and oxygen-dependent post-COVID-19 patients involved and the prevalence of women in the cohort. High BMI and comorbid pathologies such as hypertension, diabetes mellitus, and cardiovascular pathology contribute to the severity of the disease and correlate with the ACE2 blood serum level. ACE2 levels correlate with COVID-19 severity, and the concentration decreased in oxygen-dependent and critical patients compared to the mild form of the disease.

## Figures and Tables

**Table 1:** Post-COVID-19 patients in the period 1–90 days after the last negative polymerase chain reaction (PCR) test.

0–45 Days after the Last Negative PCR (59.6%)			46–90 Days after the Last Negative PCR (40.4%)	
**344**			**233**	
**Total Number**	**Females (66.9%)**	**Males (33.1%)**	**Average Age**	**Seronegative** **Patients (Control)**
577	386	191	50.63 ± 13.08 years	30
Patients based on COVID-19 severity were divided into groups				
HQM (45.6%)		HMO (37.3%)	HSV (14.2%)	HCR (2.9%)
263		215	82	17

Age. The age of patients was significantly different and increased with the severity of disease (pANOVA < 0.001, χ^2^—Pearson Chi-square test). The average age in HQM group was 44.82 ± 12.07 years, in HMO group—54.10 ± 11.88 years, HSV—58.73 ± 11.38 years, and in HCR—57.41 ± 11.41 years. According to the time after the last negative polymerase chain reaction (PCR) test, all patients were divided into two groups: (1) 0–45 days after, included 344 persons (59.6 percent), and (2) 46–90 days after, included 233 persons (40.4 percent).

**Table 2:** ACE2 level depending on COVID-19 severity (Me [Lq; Uq]) with control comparison.

Groups	ACE2, ng/mL	
	n = 440	
	n	Me [Lq; Uq]
HQM (1)	155	9.19 [8.36; 10.05]
HMO (2)	156	7.37 [5.75; 9.32]
HSV (3)	82	6.49 [5.28; 7.25]
HCR (4)	17	6.65 [5.35; 7.35]
Control group (5)	30	7.69 [6.82; 9.09]
Kruskal-Wallis test	H = 104.53; *p* < 0.001 *	
Multiple groups comparisons	p_1–2, 1–3, 1–4, 1–5_ < 0.001*, p_2–3_ < 0.05 *, p_3–5_ < 0.05 *	

Note: *—statistically significant results. Note: p_1–2_—comparison between groups HQM and HMO; p_1–3_—comparison between groups. HQM and HSV; p_1–4_—comparison between groups HQM and HCR; p_2–3_—comparison between groups HMO and HSV; p_2–4_—comparison between groups HMO and HCR; p_3–4_—comparison between groups HSV and HCR.

**Table 3:** Sex differences in ACE2 level in study groups (Me [Lq; Uq]).

		ACE2, ng/mL; n = 410	
Group		M	F
HQM (1)	n	54	101
	Me	9.05	9.22
	[Lq; Uq]	[8.19; 10.70]	[8.53; 9.94]
HMO (2)	n	60	96
	Me	7.65	6.93
	[Lq; Uq]	[6.32; 9.53]	[5.61; 9.04]
HSV (3)	n	41	41
	Me	6.67	6.05
	[Lq; Uq]	[4.95; 7.25]	[5.33; 7.17]
HCR (4)	n	9	8
	Me	6.60	7.05
	[Lq; Uq]	[5.35; 6.88]	[5.47; 7.41]
Kruskal-Wallis test		H = 41.35,	H = 60.93,
		*p* < 0.001 *	*p* < 0.001 *
Multiple groups comparisons		p_1–2, 1–3, 1–4, 2–3_ < 0.05 *	p_1–2, 1–3, 1–4_ < 0.05 *

^1^ Note. *—statistically significant results. ^2^ Note. M—males; F—females. ^3^ Note: p_1–2_—comparison between groups HQM and HMO; p_1–3_—comparison between groups HQM and HSV; p_1–4_—comparison between groups HQM and HCR; p_2–3_—comparison between groups HMO and HSV; p_2–4_—comparison between groups HMO and HCR; p_3–4_—comparison between groups HSV and HCR.

**Table 4:** ACE2 serum level (ng/mL) is a time-dependent index (Me [Lq; Uq]).

Group	0–45 Days	46–90 Days
HQM	9.41 [8.85; 10.88]	8.94 [7.43; 9.54] *
HCR	5.16 [4.75; 6.34]	6.92 [6.60; 7.41] *

Note. *—statistically significant results (*p* < 0.05).

**Table 5:** Anthropometrical parameters of persons recruited in the study depending on the COVID-19 severity.

Parameters	HQM (1)	HMO (2)	HSV (3)	HCR (4)	Statistical Significance
Weight, kg	76.02 ±17.31	83.24 # ±14.88	88.70 # ±18.04	93.41 # ±15.40	p_ANOVA_ < 0.001 * p_1–2_; p_1–3_; p_1–4_ < 0.001 *
Height, cm	166.43 ±13.35	168.25 ±9.26	168.99 ±9.67	168.76 ±9.58	p_ANOVA_ = 0.186
BMI, kg/m^2^	27.17 ±5.68	29.45 # ±4.76	31.01 # ±5.29	32.80 # ±4.91	p_ANOVA_ < 0.001 * p_1–2_; p_1–3_; p_1–4_ < 0.001 *
WC, cm	90.91 ±14.92	99.66 # ±13.49	106.63 # ±12.38	109.29 # ±9.58	p_ANOVA_ < 0.001 * p_1–2_; p_1–3_; p_1–4_ < 0.001 * p_2–3_ < 0.001 * p_2–4_ = 0.031 *
HC, cm	105.24 ±11.46	109.12 # ±9.48	111.35 # ±10.33	114.06 # ±11.71	p_ANOVA_ < 0.001 * p_1–2_; p_1–3_; p_1–4_ < 0.01 *
WHtR	0.54 ±0.09	0.59 # ±0.08	0.63 # ±0.07	0.65 # ±0.06	p_ANOVA_ < 0.001 * p_1–2_; p_1–3_; p_1–4_ < 0.001* p_2–3_ = 0.002 * p_2–4_ = 0.038 *
WHR	0.86 ±0.10	0.91 ±0.09	0.96 ±0.08	0.96 ±0.09	p_ANOVA_ < 0.001 * p_1–2_; p_1–3_; p_1–4_ < 0.001* p_2–3_ = 0.001 *
AO (WC ≥ 94 cm [M], ≥ 80 cm [W]) *	179 (69.65%)	186 (88.57%)	76 (96.20%)	17 (100.00%)	χ^2^ = 45.79; *p* < 0.001 *
AO (WHtR ≥ 0.5)	175 (67.83%)	185 (88.94%)	77 (97.47%)	17 (100.00%)	χ^2^ = 55.02; *p* < 0.001 *
AO (WHR ≥0.90 [M]; ≥0.85 [W])	137 (53.31%)	158 (75.24%)	72 (91.14%)	17 (100.00%)	χ^2^ = 58.18; *p* < 0.001 *

^1^ Note: AO—abdominal obesity; WC—waist circumference; HC—hip circumference; WHtR—waist to height ratio; WHR—waist–hip ratio; M—men; W—women. ^2^ Note: *—statistically significant results. ^3^ Note: #—statistical difference between groups HMO, HSV, HCR, and HQM (*p* < 0.05). ^4^ Note: p_1–2_—comparison between groups HQM and HMO; p_1–3_—comparison between groups HQM and HSV; p_1–4_—comparison between groups HQM and HCR; p_2–3_—comparison between groups HMO and HSV; p_2–4_—comparison between groups HMO and HCR; p_3–4_—comparison between groups HSV and HCR.

**Table 6:** ACE2 level in patients with diabetes mellitus depending on the COVID-19 severity. ACE2, ng/mL (Me [Lq; Uq]).

Group	Diabetes Mellitus		
		Absent	Present
	n	150	5
HQM (1)	Me [Lq; Uq]	9.18 [8.35; 9.9])	11.16 # [9.53; 12.77]
	n	138	18
HMO (2)	Me [Lq; Uq]	7.11 [5.57; 9.26]	8.21 # [7.19; 9.57]
	n	62	20
HSV (3)	Me [Lq; Uq]	6.20 [4.90; 7.06]	7.14 # [5.67; 8.96]
	n	13	4
HCR (4)	Me [Lq; Uq]	6.34 [4.97; 6.88]	7.37 # [7.26; 7.41]
Kruskal-Wallis test		H = 98.02 *p* < 0.001*	H = 11.92 *p* = 0.008 *
Multiple groups comparisons		p_1–2, 1–3, 1–4, 2–3_ < 0.05 *	p_1–3_ < 0.05 *

^1^ Note. *—statistically significant results compared to different severity of disease. ^2^ Note. #—statistically significant results compared to groups with absent or present diabetes mellitus. ^3^ Note: p_1–2_—comparison between groups HQM and HMO; p_1–3_—comparison between groups HQM and HSV; p_1–4_—comparison between groups HQM and HCR; p_2–3_—comparison between groups HMO and HSV; p_2–4_—comparison between groups HMO and HCR; p_3–4_—comparison between groups HSV and HCR.

**Table 7:** The time of COVID-19 diagnosis verification (admission and/or physician’s consultation and PCR testing) since the first clinical signs of disease, days.

Groups	1—HP, n = 338	2—HP + DM, n = 88	3—DM, n = 17	4—No HP, No DM, n = 500	*p* < 0.05
COVID-19 Diagnosis Verification, days	5.58 ± 0.42	4.80 ± 0.38	8.33 ± 1.60	5.01 ± 0,34	p_1–3_ = 0.037, p_2–3_ = 0.0003, p_3–4_ = 0.018

^1^ HP—Hypertension, DM—Diabetes mellitus.
